# On-Chip Construction of Multilayered Hydrogel Microtubes for Engineered Vascular-Like Microstructures

**DOI:** 10.3390/mi10120840

**Published:** 2019-12-01

**Authors:** Tao Yue, Na Liu, Yuanyuan Liu, Yan Peng, Shaorong Xie, Jun Luo, Qiang Huang, Masaru Takeuchi, Toshio Fukuda

**Affiliations:** 1 School of Mechatronic Engineering and Automation, Shanghai University, Shanghai 200444, China; yuanyuan_liu@shu.edu.cn (Y.L.); pengyan@shu.edu.cn (Y.P.); srxie@shu.edu.cn (S.X.); luojun@shu.edu.cn (J.L.); 2 School of Mechatronic Engineering, Beijing Institute of Technology, Beijing 100281, China; qhuang@bit.edu.cn (Q.H.); tofukuda@meijo-u.ac.jp (T.F.); 3 Key Laboratory of Biomimetic Robots and Systems, Ministry of Education of China, Beijing 100281, China; 4 Department of Micro-Nano Systems Engineering, Nagoya University, Nagoya 464-8603, Japan; takeuchi@mein.nagoya-u.ac.jp; 5 Faculty of Science and Engineering, Meijo University, Nagoya 468-8503, Japan

**Keywords:** microfluidic devices, on-chip fabrication, hydrogel, microfluidic assembly, multilayered microstructures

## Abstract

Multilayered and multicellular structures are indispensable for constructing functional artificial tissues. Engineered vascular-like microstructures with multiple layers are promising structures to be functionalized as artificial blood vessels. In this paper, we present an efficient method to construct multilayer microtubes embedding different microstructures based on direct fabrication and assembly inside a microfluidic device. This four-layer microfluidic device has two separate inlets for fabricating various microstructures. We have achieved alternating-layered microtubes by controlling the fabrication, flow, and assembly time of each microstructure, and as well, double-layered microtubes have been built by a two-step assembly method. Modifications of both the inner and outer layers was successfully demonstrated, and the flow conditions during the on-chip assembly were evaluated and optimized. Each microtube was successfully constructed within several minutes, showing the potential applications of the presented method for building engineered vascular-like microstructures with high efficiency.

## 1. Introduction

Cellular structures follow certain patterns and shapes inside tissues and organs, such as muscle, skin, liver, and blood vessel [1,2]. To construct artificial tissues, several important issues need to be solved, including cell encapsulating inside particular structures and high-throughput assembly of these structures [3]. Recently, as an indispensable component for building functional artificial tissues, many researchers in tissue engineering and regeneration medicine have been focusing on building engineered vascular-like structures [4,5,6]. Several construction methods for building vascular-like cell structures have been presented [7,8]. These methods include micromanipulation, microfluidics, cell proliferation control, and molding, and they have been mainly classified into cell immobilization, manipulation, and structure formation [9,10].

Convenient methods for cell immobilization are based on aspiration, non-contact force, and physical traps [11]. The large fixing force of aspiration enables its unique applications under certain conditions, while such force could damage cells [12]. Non-contact forces, such as dielectrophoresis, optical tweezers, and magnetism, have high flexibility for cell positioning, but are not suitable for long-term immobilization [13,14,15]. Using physical traps inside channels, cells can be immobilized based on certain patterns, however, the patterns are fixed, and it is difficult to transfer the cells out of the chamber for further analysis [16]. On-chip fabrication, based on photo-crosslinkable hydrogel via UV illumination, is a creative way for immobilizing cells. Such direct encapsulation of cells inside patterned hydrogels provides many advantages including high speed, low cost, and arbitrary shape [17,18,19].

Micromanipulation has been widely used to build artificial tissues [20]. Direct contact with cells can cause cell damage and contamination. Direct printing of cell-embedded microstructures and their assembly to large tissues are creative ways to build large tissue-like three-dimensional (3D) cell structures [21]. Furthermore, complex vascular structures have been directly fabricated based on 3D bioprinting technology [22,23,24,25], however, it is difficult to fit the printing resolution and accuracy for 3D structures on the dimension of small blood vessels in micron level. Nevertheless, direct formation of microtubes using microfluidic channels is possible for generating vascular-like cellular structures and networks [26,27], however, the unchangeable channel shape limits the control flexibility after the device is fabricated.

Fluidic assembly is a suitable approach for high-throughput structure formation of biological objects [28]. The 3D structures can be assembled by air [29] and complex 3D structures have been constructed by especially designed cell-laden blocks [30]. In addition, engineered biological structures have been assembled via tissue liquidity [31,32]. Tubular and cubic cellular structures have been assembled based on simple exterior stimulations and manipulations [33,34]. Furthermore, different 3D microstructures have been assembled inside microfluidic channels with particular features, which show great potential for use in assembling 3D cellular structures [35].

Beyond the structure formation, multicellular components are key elements for building functional tissue, as each type of cell has their own unique role inside the complex system of tissues [36]. For instance, several cell types can be found in the mammalian gut endothelium, including pit cells, parietal cells, zymogenic cells, and enteroendocrine cells. All these cells have defined positions and contribute to the gut endothelium in order for the complex structure to function properly [37]. A method to achieve multicellular 3D structures is becoming an important issue [38,39] and such technology would contribute significantly by revealing cell–cell interactions and functionalization factors [40]. Inside the multicellular environment, cell groups stimulate each other and form functional tissues [41,42]. Simple vascular-like microtubes have been assembled inside microfluidic channel [43]. However, these simple vascular-like hollow microtubes have contained only one type of cell, which limits their practical functionalization as artificial vascular structures. Construction of tubular structures with several different layers is a promising way to build engineered vascular-like structures, which serves the long-term target of building artificial blood vessels [44].

**Figure 1:**
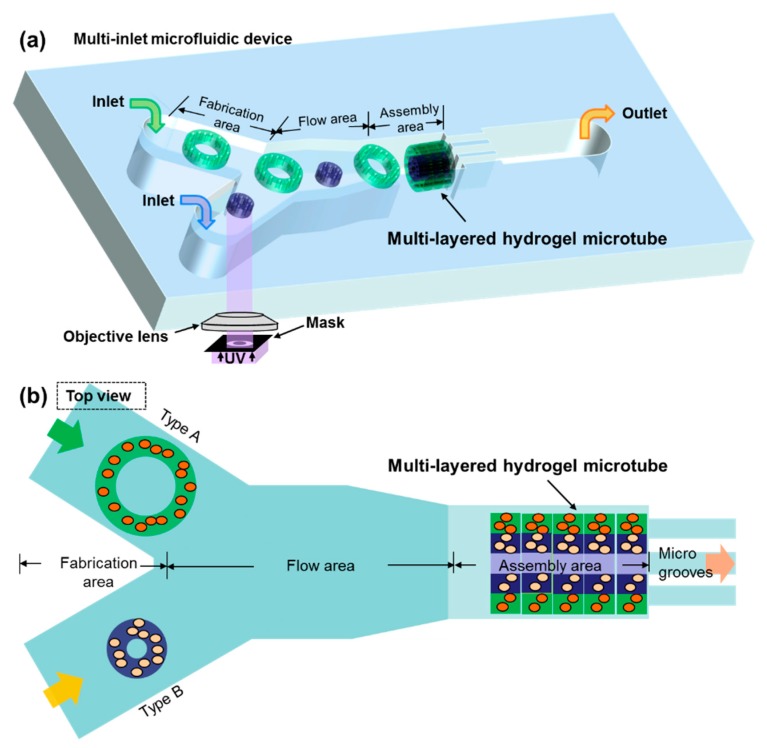
A schematic drawing of the microfluidic device for building engineered vascular-like microstructures. (**a**) The 2D microstructures are fabricated on-chip using ultraviolet (UV) light and then assembled to multilayer microtubes using the fluidic assembly approach. (**b**) Top view of the microfluidic channel consisting of fabrication, flow, and assembly areas.

In this paper, we present a novel microfluidic device for building multilayered microtubes embedding different microstructures based on fluidic assembly, as shown in Figure 1. The micro channel inside has two inlets and consists of fabrication, flow and assembly areas. On the basis of photo-crosslinkable hydrogels, an optical on-chip fabrication method has been developed to obtain two-dimensional (2D) cell embedded microstructures with arbitrary shapes. The two separately controlled inlets have been designed for fabricating 2D microstructures with different shapes or embedding different types of particles or cells. Thus, we have obtained different 2D microstructures directly in those inlets of the fabrication area, as building blocks for complex 3D structures. The flow dynamics and modification of microstructures have been experimentally evaluated. Overall, two goals have been achieved which include alternating-layered microtubes and double-layered microtubes. The experimental results of constructing both microtubes have shown potential for applications in building engineered vascular-like microstructures.

## 2. Materials and Methods

### 2.1. A Four-Layer Microfluidic Device With Two Inlets for Introducing Multiple Solutions

Multicellular structures are necessary for target tissues such as vascular-like structures. Thus, the goal is to construct vascular-like microtubes using microstructures to embed different types of particles or cells. According to the on-chip fabrication method, 2D microstructures are directly fabricated inside the channel, and the components of the microstructures depend on the prepolymer solution. One inlet can be utilized for loading one type of solution for fabrication at one time. In order to introduce multiple types of solutions into the channel at the same time, we developed a device with 2 inlets to provide the fabrication area for different types of microstructures fabricated on-chip. As shown in Figure 1, there are three different areas in the channel which are fabrication, flow, and assembly area, respectively. After the microstructures are fabricated in different inlet channels, they flow through the flow area according to the particular control procedure. The assembly area is a microwell where the microstructures flow in and rotate to vertical. To build microtubes with a diameter of 200 μm, the width and depth of the microwell is designed to be 200 μm, and the length is about 600 to 800 μm. Then the microstructures are stopped by the microgrooves one-by-one and assembled to be a microtube.

**Figure 2:**
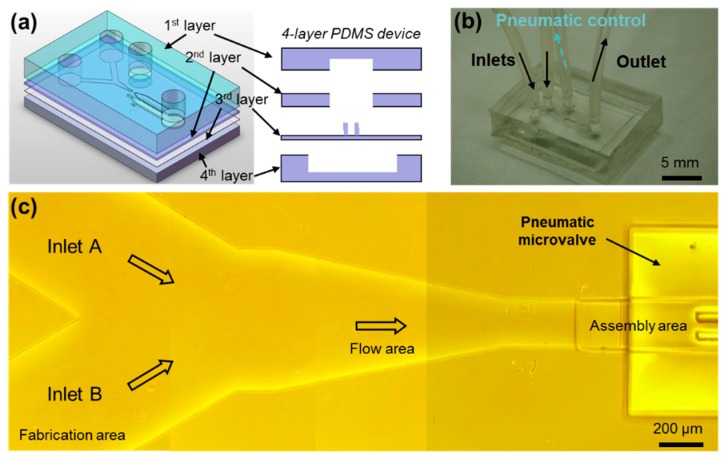
The microfluidic channel with 2 inlets for assembling multilayered microtubes. (**a**) The microfluidic device is fabrication with 4 layers of polydimethylsiloxane (PDMS). (**b**) The fabricated device for the experiments. (**c**) A micrograph of the top view of the fabricated microfluidic channel.

This microfluidic device was fabricated by four layers of polydimethylsiloxane (PDMS) based on the soft photolithography method, as shown in Figure 2a. The fabrication and flow areas were formed by the first layer. A microwell, as the assembly area, was formed by the second layer. Then, the microgrooves were fabricated on the third layer. There was a pneumatic microvalve formed by the fourth layer, in order to release the assembled microtubes by deforming the microgrooves using negative pressure. Photoresist SU-8 (SU-8 3000 series, MicroChem, Newton, MA, USA) and PDMS (SILPOT 184 W/C, Dow Corning Toray Co., Ltd., Tokyo, Japan) were used in the fabrication. The molds of the microfluidic channels were made with photoresist SU-8 by exposing ultraviolet (UV) light through the designed masks. The SU-8 molds were covered by PDMS liquid, and the PDMS was solidified after 24 h. After, the PDMS cover layers were detached from the mold, holes were made at the ends of the channels. Then the PDMS layers were bonded together. The silicone tube was connected to the holes and the solution was injected from the inlet to the channel. The fabricated 4-layer PDMS device with 2 inlets is shown in Figure 2b. A micrograph of the main channel is shown in Figure 2c. The depth of the inlet channels is 30 μm and the width of inlet channels is designed to be 500 μm to cover the size of 2D microstructures. The width and depth of the microwell is set to be 200 μm and could be modified based on the size of microtubes needed. The width of microgrooves is 30 μm to release the solution but stop the microstructures.

### 2.2. Prepolymer Solution of Photo-Crosslinkable Hydrogels

In order to demonstrate the capability of the device to fabricate and assemble different microstructures, two solutions mixed with 2 types of particles, respectively, were prepared. The main experimental solution is poly(ethylene glycol) diacrylate (PEGDA), which is a photocrosslinkable hydrogel with good biocompatibility [18,19,43]. The two prepolymer solutions, mixed by PEGDA (molecular weight 700, Sigma Aldrich, St. Louis, MI, USA), and photoinitiator (2%, Irgacure 2959, Ciba Inc., Basel, Switzerland) with two types of fluorescent microbeads (cat #17152, 0.5 μm diameter as Type A solution; cat #18660, 1 μm diameter as Type B solution) (Fluoresbrite^®^ Polysciences Inc., Warrington, PA, USA), respectively, were prepared prior to the experiments.

### 2.3. Assembly of Alternating-Layered Microtubes

**Figure 3:**
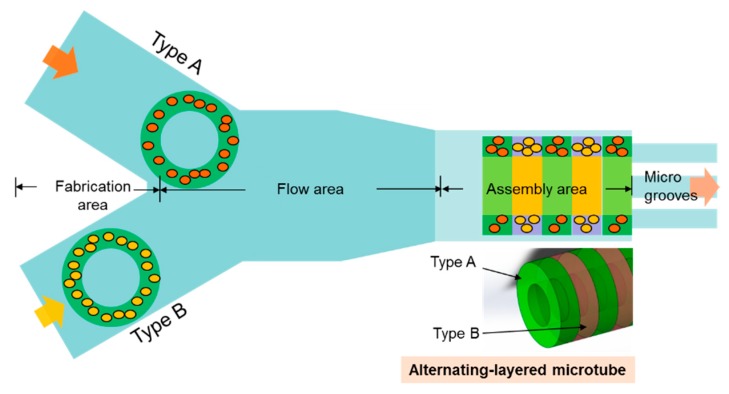
The different types of microstructures are fabricated alternately in separate fabrication areas to assemble alternating-layered microtubes in the assembly area.

As one type of valuable structures for multicellular models or cell co-culture systems, microtubes with alternating-layered structures are good examples to be fabricated. The complete fabrication method using the presented device is shown in Figure 3. Different types of microstructures with the same diameters were fabricated in each inlet, respectively. Polyethylene terephthalate (PET) masks were set between the UV lamp and the objective lens for patterning the UV light. An objective lens (40×, Olympus, Tokyo, Japan) was used for UV exposure. The UV was illuminated by a mercury lamp (UV intensity 20 mW/cm2, USH-103tems, Olympus) and the exposure time was controlled by a shutter (BSH-RIX, Sigmakoki, Tokyo, Japan). Patterned UV was exposed to the PEGDA and microstructures were fabricated inside the PEGDA solution. Microstructures were fabricated by illuminating the UV for 0.4 s. The flow was controlled by syringe pumps to make sure the microstructures in each inlet were fabricated alternately. Therefore, these two types of microstructures flowed and fell into the assembly area alternately, stopping at the microgrooves one-by-one. Finally, microtubes embedding 2 types of microstructures arranged with an alternating order were constructed.

### 2.4. Assembly of Double-Layered Microtubes

**Figure 4:**
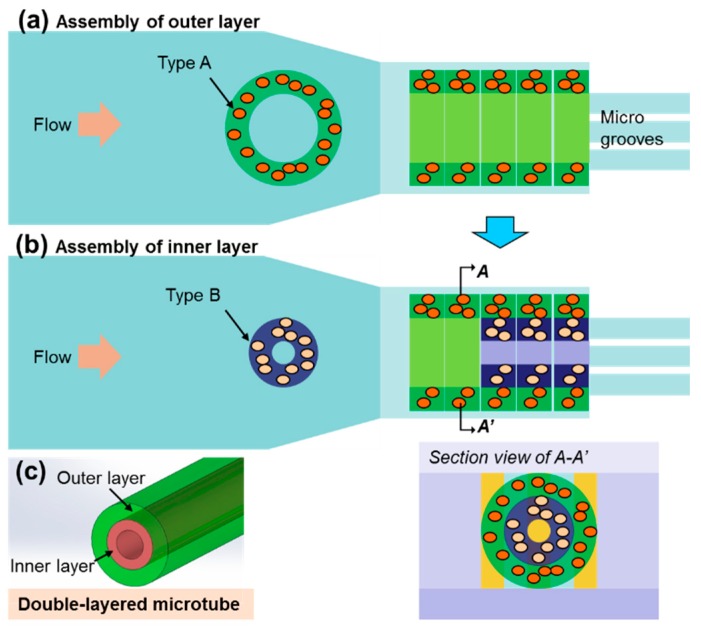
The assembly method for the double-layered microtubes, including the assembly of the outer layer as the first step (**a**) and the inner layer as the second step (**b**). (**c**) The schematic drawing of the assembled double-layered microtube.

In order to mimic the multilayered structure of vessels, a two-step assembly approach was developed to construct double-layered microtubes using the presented device. First, by introducing one type of prepolymer solution through one inlet, larger microstructures are fabricated and assembled in the microwell to be the outer layer tube, as shown in Figure 4a. Secondly, the other type of prepolymer solution was introduced by another inlet. Microstructures with smaller diameter were fabricated in this inlet and flowed into the microwell where the outer was assembled. As the outer diameter of the smaller microstructures fit the inner diameter of the outer layer tube, shown in Figure 4b, the smaller microstructures are assembled inside the outer layer tube, forming the inner layer. As shown in Figure 4c, the final microtube is a double-layered structure which has good potential to be applied as a vascular-like structure.

### 2.5. Viability Evaluation of Cells Encapsulated Inside Microstructures

Fibroblasts (NIH/3T3) were cultured inside Dulbecco’s modified Eagle’s medium (DMEM, Sigma Aldrich) with 10% fetal bovine serum (FBS, Sigma-Aldrich) for 72 h, inside an incubator (37 °C, 5% CO_2_). Prior to experiments, NIH/3T3 cells were collected and all culture medium was removed. Cells were mixed inside phosphate buffered saline (PBS, Wako, Osaka, Japan) to form a PBS cell solution of 5 × 10^6^ /mL cell concentration. The cell experimental solution contains 20% PEGDA and 0.5% photoinitiator, with a cell concentration of about 4 × 10^6^ /mL.

These cell encapsulated microstructures were washed by PBS once, and then immersed in the viability test solution, which was a mixture of 10 μL calcein AM (1 mg/mL, Wako), 15 μL propidium iodide (PI, 1 mg/mL, Wako) and 5 mL PBS. The solution and microstructures were incubated for 30 min. After that, the test solution was removed, and the microstructures were washed by PBS again. UV light (490 nm) was used to observe the samples.

### 2.6. Data Acquisition and Analysis

Bright-field and fluorescent images were obtained using an optical microscope (Olympus IX71 and CCD camera). Images of the microstructures and microtubes were processed by ImageJ (Ver. 1.47, NIH, Bethesda, MD, USA) to calculate the thickness, length, and diameter. Data are shown as mean ± standard deviation (SD) unless stated. Estimated means and standard deviation are calculated using Microsoft Excel.

## 3. Results and Discussion

### 3.1. Alternating-Layered Microtubes are Assembled by Controlling the Fabrication, Flow, and Assembling Sequences On-Chip

**Figure 5:**
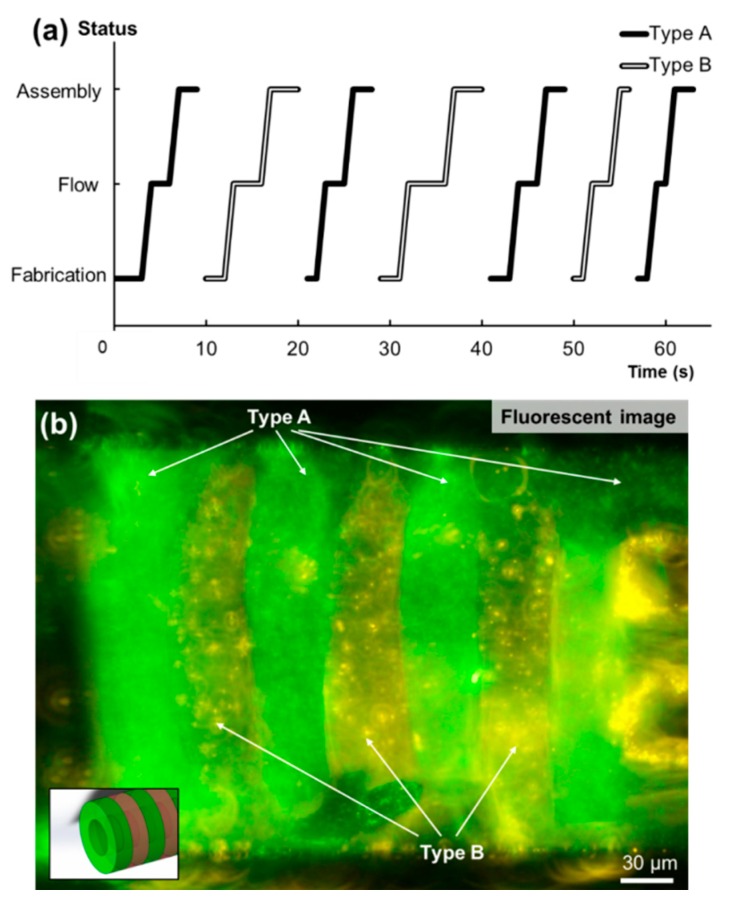
(**a**) The alternating-layered microtubes are assembled by controlling the fabrication, flow, and assembly time of the two types of microstructures in the channel. (**b**) The fluorescent image of the assembled microtube with alternating layers.

Aiming to construct alternating-layered microtubes, two types of microstructures were fabricated in different inlets and assembled based on a particular control sequence. The key is to fabricate, transport, and assemble these two types of microstructures with desired orders. An assembly sequence was tested based on the two syringe pumps connected to the inlets, as shown in Figure 5a. There were fabrication, flow and assembly times according to the three areas of the channel. The data shows the different status of each microstructure during the whole assembly process, from the first structure to the seventh by each polygonal line. It took about 10 s to complete the whole assembly process for each microstructure. After assembly, the solution in the channel was replaced to Deionized (DI) water. Some PEGDA still remained on the surface of the fabricated microstructures after solution replacement. These PEGDA were not totally crosslinked and able to conduct a secondary crosslinking for connecting the assembled layers. A secondary UV exposure for about 1 second was given to the whole assembled structure which made the assembled layers firmly connected. Finally, a microtube, embedding seven layers of two types of microstructures which were arranged in an alternating order, was built.

Here, fluorescent beads were used to indicate and show the alternating structures and demonstrate the construction sequence, as shown in Figure 5b. The green layers and yellow layers represent two types of microstructures. After the assembly, the tube was extracted directly from the channel using the pneumatic microvalve under the assembly area. Then, new microstructures were fabricated, and the next tube was assembled without changing or cleaning the device. The success rate of the assembly was high, at about 90%, and the whole process was efficient as a “micro-factory assembly line” inside the channel. These alternating-layered microtubes are ideal platforms to encapsulate multiple types of cells close to each other. Furthermore, the sequence and shape of the assembled 2D microstructures can be changed according to different purposes, and therefore could be applied for investigating cell–cell interaction and cell proliferation inside such 3D cell culture environment.

### 3.2. Optimized Diameter of Inner Layer Structures was Obtained for Assembling Double-Layered Microtubes

**Figure 6:**
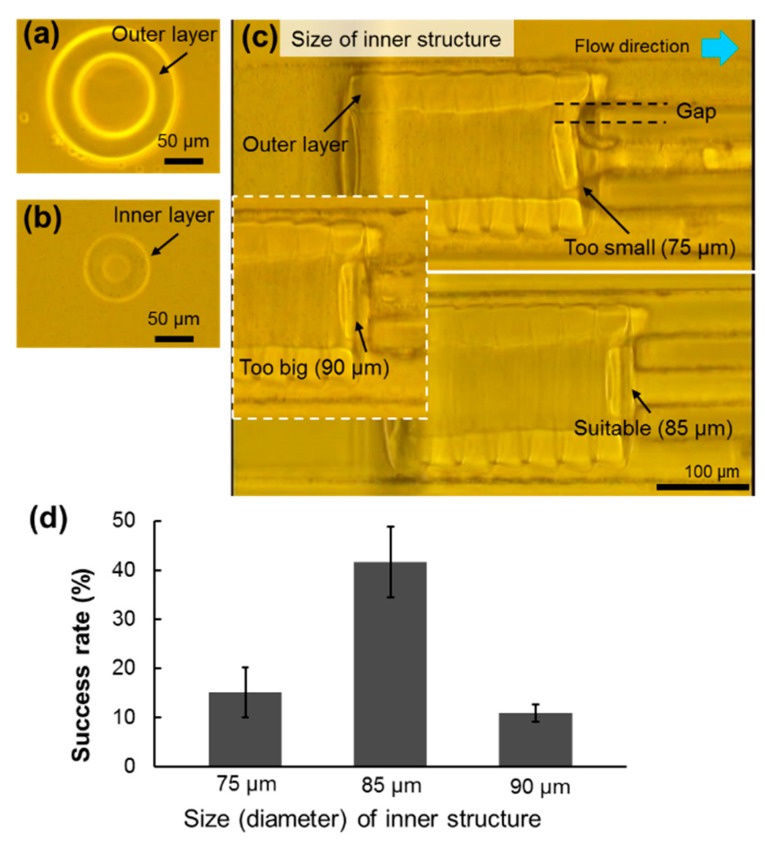
Evaluation of the size of inner structures for constructing double-layered microtubes. (**a**,**b**) The two fabricated types of donut microstructures for the outer and inner layers, respectively. (**c**) The different inner structures are tested to select the optimized size for the assembly inside the outer layer. (**d**) The assembly success rate for inner structures with different size. All data are presented as the mean ± SD, *n* = 10.

To construct double-layered microtubes, microstructures with different dimensions of outer and inner layers were fabricated in different inlets, respectively. In the experiments, the diameter of the outer layer was designed to be the same as the width of the assembly area and the microwell, making the gap between them as small as possible for assembling the inner layer. The fabricated microstructure for the outer layer is shown in Figure 6a, with about 190 μm in the outer diameter and 95 μm in the inner diameter. Figure 6b shows one example of microstructures for the inner layer. Because the inner layer microstructures need to flow inside the hollow of the outer layer tube, the outer diameter of the inner structures becomes very important and influences the final assembly results significantly. Therefore, three types of inner structures were tested and evaluated, as shown in Figure 6c. Their diameters were about 75 μm, 85 μm and 90 μm, respectively, in order to optimize the dimensions of inner microstructures.

The smaller microstructures flowed into the hollow of the outer layer and assembled inside. The assembly results with the three different diameters were evaluated. The definition of a successful assembly of the inner layer is that the microstructure rotates 90° inside the hollow of outer layer, and stops at the end of the hollow, which is also the entrance of the microgrooves. As the results shown in Figure 6c, the inner structure rotated 90°, flowed into the hollow and attached at the microgrooves, which was a successful case. Other results are defined as failure cases, such as flow through the groove (size is too small), block outside the hollow (size is too big), and rotation failure. The gap between the outer layer and inner layer should not be too big, otherwise the connection between two layers is bad, as shown by the 75 μm case. However, if the inner microstructures are too big, they could be difficult to flow into the outer layer, as the 90 μm case represented. The assembly results are evaluated, as shown in Figure 6d. The assembly success rate was defined as the percentage of successful cases in the total assembly cases.

On the basis of these assembly experiments, the assembly success rate of the 75 μm case was low because such small inner microstructures could easily leak out into the microgrooves. In addition, although sometimes it was assembled successfully, the gap between the outer layer and inner layer was too large. In the 90 μm case, the assembly success rate was also low because it was difficult for the large inner microstructures to flow into the hollow of the outer layer. The 85 μm case showed better assembly results that were about 25% higher in success rate than other cases. It also has a suitable gap between the inner layer and outer layer for assembling double-layered microtubes. Therefore, the 85 μm diameter of inner layer was utilized in the following experiments.

### 3.3. Square-Shaped Outer Layer Microstructures were UtiLized to Modify the Flow Conditions for Improving the Assembly of Double-Layered Microtubes

**Figure 7:**
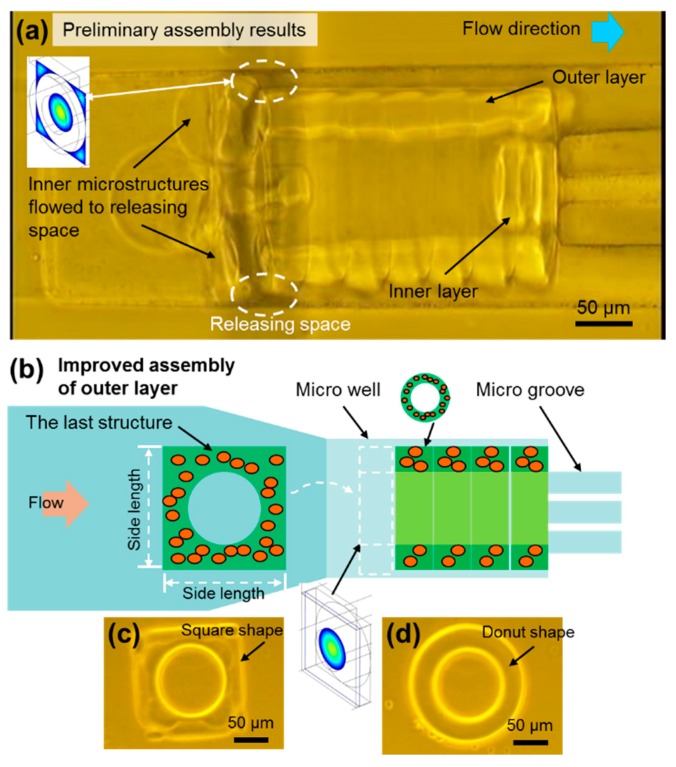
Flow condition modification using outer layer structures for double-layered microtubes. (**a**) The assembly of the inner layer in the cylinder-shaped outer layer. Preliminary results for the assembly show the inner layer microstructures blocked at the entrance of the hollow because of the releasing space at the corners. (**b**) The schematic drawing of the square-shape microstructure assembling as the final block of the modified outer layer. This square-shaped microstructure will improve the assembly results. (**c**) A micrograph of the square-shaped microstructure. (**d**) A micrograph of the donut-shaped microstructure that is used to build the outer layer.

The assembly of each microstructure is performed based on an axis-transformed fluidic assembly inside the microwell. However, the flow condition is different for the inner layer assembly as compared to the outer layer. While one of the key parameters is the diameter of the inner structures, described previously, another factor is that the flow condition changes after the outer layer has been assembled inside the microwell. Figure 7a shows a preliminary assembly result and there are some spaces between the channel wall and the cylinder-shaped outer layer, called “the releasing space”. The assembly of inner layers is unstable because of the releasing space. The microstructures for the inner layer can flow to the releasing space following the solution, or flow into the hollow as we want. However, after the first inner microstructure is assembled inside the hollow of the outer layer, the flow rate inside the hollow is decreased, and correspondingly the flow rate in the releasing space is relatively increased. This means the inner microstructures go into the releasing space and cause more assembly failures.

As shown in Figure 7a, there were many inner microstructures that flowed into the releasing space and blocked the entrance of the hollow. It was difficult for the microstructures of the inner layer to flow into the hollow of the outer layer, because of these assembly failures. The inner structures failed to flow into the hollow of the outer layer and were blocked outside. This resulted in a bad assembly with about 40% success rate, as shown for the 85 μm case in Figure 6d. Therefore, modification of the releasing space and improvement of the flow condition for assembling the inner layer are needed.

In order to solve this problem, the releasing space should be blocked to let as much flow as possible go into the hollow of the outer layer. Figure 7b shows a simple method to achieve this. The last structure of the outer layer is changed to a square-shaped structure. In this case, the square-shaped structure blocks the space between the channel wall and the outer layer, making the inner layer structures flow into the hollow more smoothly. The square-shaped microstructure is shown in Figure 7c and its side length is the same as the diameter of outer layer, fitting the size of the microwell. Theoretically, the releasing space between outer layer and the channel wall would be blocked.

On the basis of the improvement, the flow velocity in the hollow of the outer layer was increased, which could be easily calculated based on the relationship between the flow velocity, *v*, and cross-sectional area, *A*, through which the flow passes, under constant flow rate, *Q*.

*Q* = *v* · *A* (1)

Regarding experimental parameters, the outer diameter of the outer layer is 200 μm, and the inner diameter is 90 μm. Before and after the modification, the change of *A* can be calculated, and, then, the increase of *v* can be obtained. The modified *v* is approximately 2.3 times higher than the original value. This indicates an increase of flow velocity inside the hollow and relates with the higher possibility of the inner layer structures flowing into the hollow. Figure S1 shows the simulation results of the modified case. It is clear that the flow only goes into the hollow of the outer layer during the assembly of the inner layer. The inner layer structures flow with the solution into the hollow, resulting in a higher assembly success rate of the inner layer than before.

### 3.4. High-Efficient Assembly of Double-Layered Microtubes as Engineered Vascular-Like Microstructures

**Figure 8:**
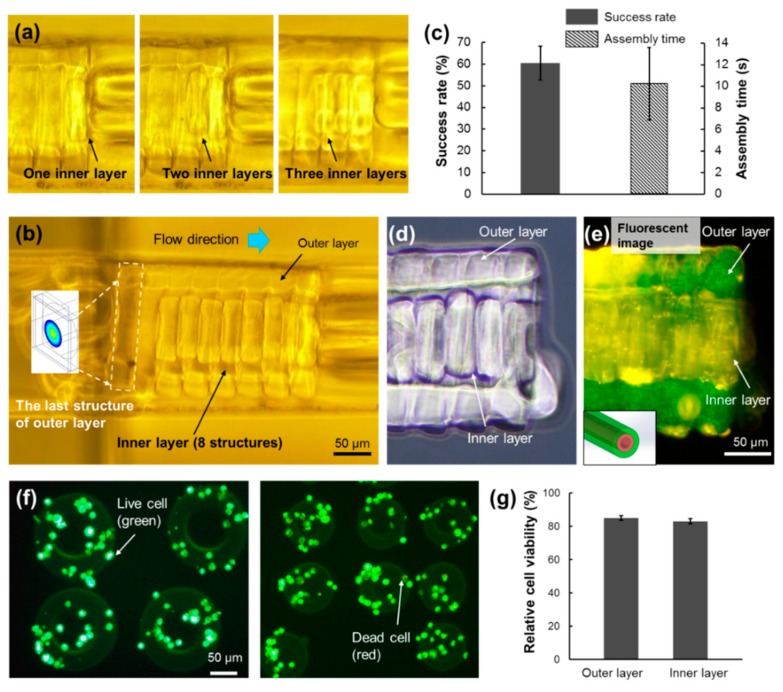
The assembly results of the double-layered microtubes. (**a**) The assembly of the first 3 structures of the inner layer. (**b**) The assembled double-layered microtube with 8 structures in the inner layer. The square-shaped microstructure is the last building block of the modified outer layer. (**c**) The assembly success rate and the average assembly time for one structure of the inner layer. All data are presented as the mean ± SD, *n* = 10. The outer layer and the inner layer are clearly observed in the bright field image (**d**) as well as the fluorescent image (**e**) of the double-layered microtube. (**f**) Cell viability tests are conducted for both types of microstructures of outer layer and inner layer. (**g**) The relative cell viability of all the tested samples is higher than 80%.

On the basis of the suitable diameter of the inner layer and the modified outer layer structure, double-layered microtubes have been constructed with high efficiency as shown in Figure 8a. After the first inner layer was assembled, the flow rate inside the outer layer hollow decreased. In addition, the real width of the microwell in the experiment was not exactly the same as the diameter of the outer layer. Actually, it was a little bit larger than the outer layer for conducting the outer layer assembly. Therefore, a small gap remained between the microwell and the outer layer, and there was a small flow that went out of the hollow. During the assembly of the inner layer, some inner microstructures were blocked outside the entrance of the outer layer, as shown in Figure 8b. However, at least eight layers of the inner layer assembly succeeded and double-layered microtubes were built. Compared to the former assembly result, which was only four successful structures with a low success rate, the modified cases were much better.

The assembly success rate and the average assembly time for one inner structure are shown in Figure 8c. To assemble one piece of the inner microstructure, it took about 10 s for the whole process. It took about 2 min to build this microtube containing eight layers. Although there were still inner layers blocked outside the hollow, the assembly success rate was improved to about 60%, which was 20% higher than the preliminary assembly without the square outer microstructure. This assembly result demonstrated that this two-step assembly method for building double-layered microtubes was feasible. Furthermore, the advantages of on-chip fabrication and microfluidic assembly, such as short operating time and control-free environment, have highly contributed to the construction of double-layered microtubes.

During the experiments, fluorescent beads were used to identify the outer layer and inner layer. After assembly, the microtube was extracted from the channel using the pneumatic microvalve and observed outside the channel. Figure 8d,e clearly shows both the outer layer and the inner layer in the microtube. According to the embedded fluorescent microbeads, it clearly shows different colors for the outer layer, which is green, and the inner layer, which is yellow. As mentioned in Figure 8c, there is still a small flow out of the hollow of the outer layer during the assembly of the inner layer, which brought the yellow fluorescent beads to adhere to the outside surface of the outer layer and the small parts of the outer layer became yellow, as shown in the top-right region of Figure 8e.

In order to confirm that the presented method could be used in biological applications, cell viability evaluations were conducted after the cells were encapsulated inside the microstructures. Fluorescent images of the evaluation results are shown as Figure 8f. The green represents live cells, whereas the red represents dead cells. The two different microstructures for the outer layer and inner layer were both tested. Figure 8g shows that the relative cell viability of all the samples is higher than 80%, which confirms that they are able to be applied for biological applications.

One thing that should be noted is the usage of UV light and radical photoinitiator. Although the UV exposure time is short and the photoinitiator concentration is low, they limit the type of cells that can be used. For example, stem cells could be strongly influenced in terms of differentiation and maturation. However, PEGDA is not biodegradable, which means PEGDA remains in the constructed microtubes even after long-term culture. Therefore, other alternative biodegradable hydrogels, such as gelatin methacrylate (GelMA), were also tested in the presented device. The remaining issue is that fabricated GelMA microstructures are stuck on the channel bottom, which makes further fluidic assembly impossible. Surface treatments for PDMS (such as ultrahydrophobic surface) could possibly solve this problem and prevent the adhesion of GelMA on the PDMS surface.

An ultimate goal is to achieve blood vessels, such as venules, which mainly have three layers. The inner layer is a single layer of endothelial cells glued by a polysaccharide intercellular matrix. This layer is built by continual flow with the HUVECs inside the engineered microtubes [44]. The middle layer is rich in vascular smooth muscle cells and elastic tissues, which control the caliber of the vessel. The outer layer is entirely made of fibrous connective tissue. Therefore, at least the engineered outer and middle layers are needed for constructing functional vascular-like tissues. This makes our double-layered microtubes feasible for future applications in mimicking real vessel structures for functional engineered vascular-like structures.

## 4. Conclusions

We presented a new method to construct multilayered hydrogel microtubes by direct fabrication and assembly inside microfluidic channels. A four-layered microfluidic device with two inlets for constructing such microtubes was developed. Two goals have been achieved, including alternating-layered microtubes and double-layered microtubes. Alternating-layered microtubes were constructed by controlling the fabrication, flow, and assembly time of each microstructure. Double-layered microtubes were successfully demonstrated with high efficiency, based on the improvement of both inner and outer layers, as well as the optimization of flow conditions during the flow and assembly. As a future application, different types of cells could be utilized in the presented microfluidic device to build multicellular microtubes as functional in vitro engineered vascular-like structures.

## Supplementary Materials

The following are available online at https://www.mdpi.com/2072-666X/10/12/840/s1, Figure S1: The simulation for the flow condition inside the channel after the outer layer was assembled.
