# Supplementary material for: On-Chip Construction of Multilayered Hydrogel Microtubes for Engineered Vascular-Like Microstructures
Source: Micromachines (Basel). 2019 Dec 1;10(12):840. doi: 10.3390/mi10120840 (PMC6953073; doi:10.3390/mi10120840)
Supplement: Supplementary file 1 [file micromachines-10-00840-s001.pdf]

Supplementary Materials

On-Chip Construction of Multi-layered Hydrogel Microtubes for Engineered Vascular-Like Microstructures

Tao Yue Na Liu, Yuanyuan Liu, Yan Peng, Shaorong Xie, Jun Luo, Qiang Huang, Masaru Takeuchi and Toshio Fukuda

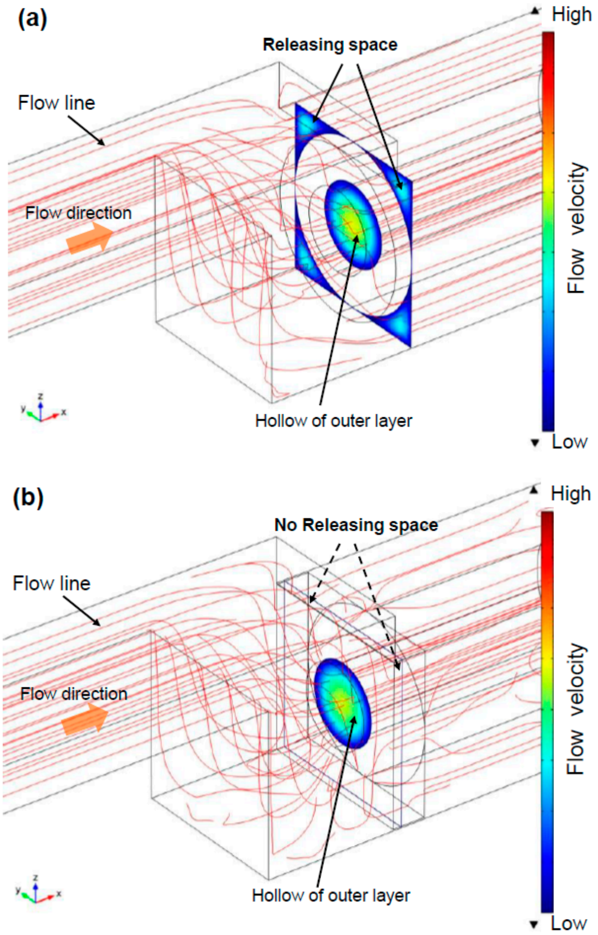

Figure S1. (a) The simulation for the flow condition inside the channel after the cylinder outer layer was assembled by all donut shaped microstructures. As observed in preliminary experiments, after outer layer is assembled, the flow condition for inner layer structures is changed. The flow went not only into the hollow of outer layer, but also the releasing space between the channel wall and the outer layer, which influenced the assembly of inner layer. **(b)** The simulation for the flow condition inside the channel after the assembly of the square shaped microstructure for outer layer as the last building block, making the flow condition more stable for assembling the inner structures inside the outer layer.

© 2019 by the authors. Licensee MDPI, Basel, Switzerland. This article is an open access article distributed under the terms and conditions of the Creative Commons Attribution (CC BY) license (http://creativecommons.org/licenses/by/4.0/).
